# Optimization of Acquisition time of ^68^Ga-PSMA-Ligand PET/MRI in Patients with Local and Metastatic Prostate Cancer

**DOI:** 10.1371/journal.pone.0164392

**Published:** 2016-10-18

**Authors:** Susanne Lütje, Sebastian Blex, Benedikt Gomez, Benedikt M. Schaarschmidt, Lale Umutlu, Michael Forsting, Walter Jentzen, Andreas Bockisch, Thorsten D. Poeppel, Axel Wetter

**Affiliations:** 1 Clinic for Nuclear Medicine, University Hospital Essen, Hufelandstraße 55, 45122 Essen, Germany; 2 Department of Diagnostic and Interventional Radiology and Neuroradiology, University Hospital Essen, Hufelandstraße 55, 45122 Essen, Germany; 3 University Dusseldorf, Medical Faculty, Department of Diagnostic and Interventional Radiology, Moorenstraße 5, 40225 Dusseldorf, Germany; University of South Australia, AUSTRALIA

## Abstract

**Objective:**

The aim of this optimization study was to minimize the acquisition time of ^68^Ga-HBED-CC-PSMA positron emission tomography/magnetic resonance imaging (PET/MRI) in patients with local and metastatic prostate cancer (PCa) to obtain a sufficient image quality and quantification accuracy without any appreciable loss.

**Methods:**

Twenty patients with PCa were administered intravenously with the ^68^Ga-HBED-CC-PSMA ligand (mean activity 99 MBq/patient, range 76–148 MBq) and subsequently underwent PET/MRI at, on average, 168 min (range 77–320 min) after injection. PET and MR imaging data were acquired simultaneously. PET acquisition was performed in list mode and PET images were reconstructed at different time intervals (1, 2, 4, 6, 8, and 10 min). Data were analyzed regarding radiotracer uptake in tumors and muscle tissue and PET image quality. Tumor uptake was quantified in terms of the maximum and mean standardized uptake value (SUV_max_, SUV_mean_) within a spherical volume of interest (VOI). Reference VOIs were drawn in the gluteus maximus muscle on the right side. PET image quality was evaluated by experienced nuclear physicians/radiologists using a five-point ordinal scale from 5–1 (excellent—insufficient).

**Results:**

Lesion detectability linearly increased with increasing acquisition times, reaching its maximum at PET acquisition times of 4 min. At this image acquisition time, tumor lesions in 19/20 (95%) patients were detected. PET image quality showed a positive correlation with increasing acquisition time, reaching a plateau at 4–6 min image acquisition. Both SUV_max_ and SUV_mean_ correlated inversely with acquisition time and reached a plateau at acquisition times after 4 min.

**Conclusion:**

In the applied image acquisition settings, the optimal acquisition time of ^68^Ga-PSMA-ligand PET/MRI in patients with local and metastatic PCa was identified to be 4 min per bed position. At this acquisition time, PET image quality and lesion detectability reach a maximum while SUV_max_ and SUV_mean_ do not change significantly beyond this time point.

## Introduction

Prostate cancer (PCa) causes significant morbidity and accounts for a tremendous amount of cancer-related deaths in men. During the past decade, radionuclide imaging techniques such as ^11^C- of ^18^F-choline based positron emission tomography/computed tomography (PET/CT) have attracted attention as these techniques allow sensitive diagnosis of PCa in early stages of primary PCa and metastatic disease as well as disease recurrence. However, the use of choline as a tracer for PET/CT is restricted by limited sensitivity for the detection of PCa in patients with serum prostate-specific antigen (PSA) levels of < 2 ng/ml [1–4]. To address this limitation, new tracers which allow more sensitive and specific detection of PCa with PET have been developed, such as ligands of the prostate-specific membrane antigen (PSMA) [5].

PSMA is a type II integral membrane glycoprotein which was first detected on the human prostatic carcinoma cell line LNCaP [6]. In malignant tissue, increased PSMA expression was found to be expressed in the stroma adjacent to neovasculature of solid tumors, suggesting PSMA to be involved in angiogenesis [7]. Due to its selective overexpression in 90–100% of primary PCa lesions, malignant lymph nodes, and bone metastases [8–10], PSMA is considered a reliable tissue marker for PCa and an ideal target for theranostic applications [11–15].

Recently, highly specific PSMA ligands such as ^68^Ga-labeled HBED-CC-PSMA or ^18^F-labeled DCFPyl have been developed and clinically tested, showing promising results for the detection of PCa lesions with PET/CT [16–18]. However, the use of diagnostic full-dose CT scans is accompanied with intravenous administration of contrast agents, which might restrict the use of PET/CT in patients with impaired kidney function and where multiple follow-up examinations are needed. Magnetic resonance imaging (MRI), in contrast, does not involve ionizing radiation. In addition, despite the successful implementation of these tracers in PET/CT imaging, exact morphological tumor staging of the prostate is not possible in CT. In integrated PET/MRI, high quality prostate imaging is possible during PET data acquisition. Furthermore, first studies indicate that PET/MRI is superior to PET/CT in the detection of bone metastases, leading to a more accurate tumor staging in a true “one stop shop” examination [19, 20]. For these reasons, hybrid whole-body PET/MRI scanners is expected to be useful for the detection of PCa lesions.

So far, the feasibility of ^68^Ga-HBED-CC-PSMA PET/MRI for the detection of recurrent PCa has been evaluated in one study [21]. Preliminary results suggest that PCa could be detected more easily and more accurately with ^68^Ga-HBED-CC-PSMA PET/MRI as compared to PET/CT.

In contrast to PET/CT imaging, PET data acquisition in PET/MRI is performed simultaneously to the time-consuming MR data acquisition, allowing variations in the duration of acquisition time in integrated PET/MRI systems. By affecting count statistics and image noise, the PET acquisition and the administered activity are the key factors determining PET image quality and PET quantification accuracy and represent essential parameters in forthcoming PET/MRI protocols. So far, the optimal PET acquisition time for optimal image quality and diagnostic accuracy in PET/MR imaging using PSMA ligands has not been identified.

Therefore, the aim of this study was to evaluate the role of variations in PET acquisition time for image quality and SUV_max_/SUV_mean_ and thus improve the imaging protocols of ^68^Ga-HBED-CC-PSMA PET/MRI in patients with local and metastatic prostate cancer.

## Materials and Methods

### Patient characteristics

This study was approved by the local ethics committee (Ethics committee University Hospital Essen, Essen, Germany) as part of a general ethical approval on PET/MRI research. Written informed consent was obtained from all patients. Twenty patients with primary (n = 10) or metastatic PCa (n = 10) were included into this retrospective study. The average age of the patients was 69 years (range 55–82 years) and their median serum PSA level was 19.0 ng/ml (range 0.9–161.0 ng/ml). Patient characteristics are summarized in **Table 1**.

**Table 1:** Patient characteristics.

Patient no	Age (years)	PSA (ng/ml)	Activity (MBq)	Timepoint p.i. (min)	Type (primary/recurrent)	Prostate/ prostate bed lesion	LN lesion	Bone lesion
1	56	12.0	108	77	primary	1	0	0
2	82	3.1	135	153	recurrent	1	0	0
3	79	3.7	140	113	recurrent	1	2	0
4	61	4.5	92	149	recurrent	1	0	0
5	72	0.9	149	121	recurrent	0	2	0
6	73	12.9	119	119	primary	1	0	0
7	64	35.0	97	106	primary	1	0	0
8	63	4.4	147	179	recurrent	1	2	0
9	55	1.1	133	192	recurrent	0	2	1
10	62	5.3	85	181	primary	1	0	0
11	69	10.0	76	320	primary	1	0	0
12	76	48.0	93	154	primary	1	1	2
13	76	6.4	110	210	recurrent	1	9	0
14	66	2.3	146	134	recurrent	0	1	0
15	65	4.9	148	242	primary	1	0	0
16	76	4.7	149	153	recurrent	0	0	2
17	73	10.0	119	200	recurrent	0	3	0
18	81	161.0	129	169	primary	1	>10	0
19	67	26.0	145	219	primary	1	0	0
20	65	23.5	101	180	primary	1	0	1

### Radiolabeling

^68^Ga^3+^ was obtained from a ^68^Ge/^68^Ga radionuclide generator (Isotopen Technologies Garching GmbH) and complexed with the HBED-CC conjugate as described previously [21]. The ^68^Ga-labeled HBED-CC conjugate of the PSMA-specific pharmacophore Glu-NH-CO-NH-Lys was synthesized as described previously [21]. The radiolabeling and purification of the PSMA ligand was performed using an automated module (Ga-68 Cassette Labeling Module GAIA by Raytest). All preparations contained 10 μg of the PSMA ligand. All dosages were measured prior to injection.

### PET/MR imaging

The ^68^Ga-HBED-CC-PSMA ligand was administered intravenously to the patients (mean activity 99 MBq/patient, range 76–148 MBq). Variations in injected radiotracer activity were a consequence of the relatively short half-life of ^68^Ga. All preparations contained 10 μg of the PSMA ligand. All activities were measured prior to injection.

At, on average, 168 min (range 77–320 min) after administration of ^68^Ga-HBED-CC-PSMA (mean 121 MBq/patient, range 76–149 MBq), whole-body or pelvic PET/MR imaging was performed on a Biograph mMR (Siemens AG, Healthcare Sector, Erlangen, Germany). All patients primarily underwent a whole-body or pelvic 3D-volume interpolated breath-hold examination (VIBE) sequence (TR 3.6 ms, TE1 1.23 ms, TE2 2.46 ms, slice thickness 3.12 mm, FOV 500 mm) in the Dixon technique for MR-based scatter correction. In addition, several other MR sequences were conducted. An overview of these MR sequences is provided in **Table 2**. PET/MR imaging of the pelvic area as well as the whole body was performed with 4 min per bed position. The entire examination took approximately 1 hour per patient. PET images were reconstructed using an iterative reconstruction algorithm (3 iterations, 21 subsets) with a 3D Gaussian filter of 4 mm. Time frames used in the image reconstruction were 1, 2, 3, 4, 6, 8 and 10 min derived from the list mode data. Random-, scatter-, and decay correction was applied for all emission data.

**Table 2:** MR imaging sequence parameters.

Sequence	TR (ms)	TE (ms)	FoV (mm)	Slice thickness (mm)	Matrix	B-values (s/mm^2^)	Voxelsize
TIRM coronal	3110	56	380 80.4	5	448 75		0.8x0.8x5mm
T1 FSE axial	445	9.6	40068.8	7	512 56		0.8x0.8x7mm
T2 FSE axial	4311	114	40068.8	7	512 56		0.8x0.8x7mm
T2 FSE axial	4320	101	200100	3	320 97		0.6x0.6x3mm
T2 FSE coronal	4000	101	200100	3	320 97		0.6x0.6x3mm
T2 FSE sagittal	3740	101	200100	3	320 97		0.6x0.6x3mm
T1 fs axial	808	11	200100	2	512 56		0.4x0.4x2mm
T1 vibe fs axial	4.41	2.15	42075	3	512 56		0.9x0.9x3mm
T1 fs transversal (contrast)	606	10	40068.8	7	512 56		0.8x0.8x7mm
T1 vibe dyn (contrast)	4.01	1.31	30078.7	4	192 76		2x1.5x4mm
DWI	9600	93	260	3.6	160	0, 800, 1000	1.6x1.6x3.6mm

### Image analysis

#### Qualitative analysis

The qualitative analysis was performed patient- and lesion-based. The images acquired by the PET component of the PET/MRI system were initially categorized upon the presence of tumor lesions based on visual evaluation and if present, into soft-tissue lesions originating from the prostate gland, lymph node lesions or bone lesions. In analogy to a recent publication, image quality was evaluated visually based on a five-point ordinal scale ranging from 5–1 (excellent—insufficient) [22]. All data sets were analyzed by two board-certified specialists in nuclear medicine and radiology independently. Any disagreements were resolved by consensus. Data analysis was performed using syngo.via software (Siemens AG, Healthcare Sector, Erlangen, Germany). Only clearly identifiable tumors on PET were evaluated.

#### Quantitative analysis

Uptake of the ^68^Ga-HBED-CC-PSMA ligand in tumor lesions were quantified regarding their maximum and mean standardized uptake values (SUV_max_ and SUV_mean_) as it is standard in clinical routine. SUVs were calculated by drawing spherical volume of interest (VOI) regions around areas with focally increased uptake on transverse slices and automatically adapted to a 3-D volume of interest thresholded at a 40% isocontour. Reference VOIs were drawn in the gluteal muscles on the right side to determine background tracer accumulation and tumor-to-background ratios. The native SUVs of the lesions were normalized to the SUVs in the reference region.

### Statistical analysis

Statistical analyses were performed with Graphpad Prism, version 6 (GraphPad, La Jolla, USA). Results are presented as mean ± standard deviation (SD). Descriptive statistics were used for patients’ characteristics and SUV_max_. PET image quality and SUV_max_/SUV_mean_ of primary vs. recurrent and prostate/prostate bed vs. lymph node lesions of PCa were compared by Mann Whitney U tests.

## Results

### Patients

At the reference standard of 10 min of acquisition time, in all 20 patients, at least one tumor lesion was detected (**Table 2**). Ten out of 20 patients had primary PCa, while all other patients had recurrent disease. Out of the patient group with primary PCa, 7/10 (70%) had a tumor lesion solely within the prostate and 3/10 (30%) had tumor manifestations in the prostate and lymph nodes (n = 2) or in the prostate and bones (n = 1). In the group of patients with recurrent PCa, tumor lesions were located solely within the prostate/prostate bed (2/10), lymph nodes (3/10), and bones (1/10). The remaining patients with recurrent disease had tumor manifestations at multiple locations out of these categories.

### Tumor lesion detectability

**Fig 1:**
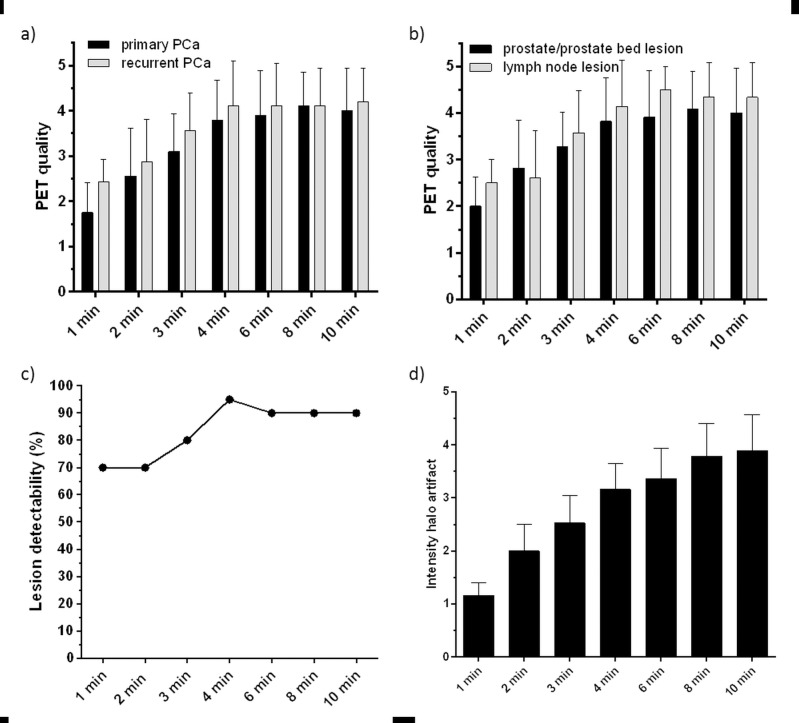
Quality of PET images obtained with ^68^Ga-HBED-CC-PSMA PET/MRI at different acquisition times for primary (n = 10) and recurrent (n = 10) PCa lesions (a)) and prostate/prostate bed as well as lymph node lesions (b)), data are presented in mean ± SD. c) Percentage of tumor lesions detected with ^68^Ga-PSMA PET/MRI at different acquisition times. d) Intensity of the halo artifact on an ordinal scale from 1 (not present)– 5 (very intense halo artifact) at different PET image acquisition times. Data are presented in mean ± 95% confidence interval.

Lesion detectability linearly increased with increasing acquisition times, reaching its maximum at PET acquisition times of 4 min. At this image acquisition time, tumor lesions in 19/20 (95%) patients were detected. At PET acquisition times of 1, 2, and 3 min, tumor lesions were detected in 14/20, 14/20, and 16/20 patients, respectively (**Fig 1**). At image acquisition times of 6, 8, and 10 min, lesion detectability slightly decreased to 90% (18/20 patients), which was caused by reduced PET signal intensity around the urinary bladder obscuring the tumor lesion.

### Qualitative analysis

PET image quality showed a linear correlation with increasing acquisition time, reaching a plateau at 4 min of image acquisition for both patients with primary as well as for patients with recurrent PCa (**Fig 1**). On the ordinal image quality scale ranging from 5 (excellent) to 1 (insufficient), the mean PET quality was 2.1 ± 0.7 for 1 min, 2.7 ± 1.0 for 2 min, 3.3 ± 0.9 for 3 min, 3.9 ± 0.9 for 4 min, 4.0 ± 1.0 for 6 min, 4.1 ± 0.8 for 8 min, and 4.1 ± 0.9 for 10 min. Mean PET image quality for tumor lesions in patients with primary PCa did not differ significantly from that of lesions in patients with recurrent PCa. PET image quality of lesions within the prostate and prostate bed showed the same trend, reaching image qualities of 2.0 ± 0.6 for 1 min, 2.8 ± 1.0 for 2 min, 3.3 ± 0.7 for 3 min, 3.8 ± 0.9 for 4 min, 3.9 ± 1.0 for 6 min, 4.1 ± 0.8 for 8 min, and 4.0 ± 1.0 for 10 min (n = 11 patients). PET image quality of lesions within lymph nodes showed the same trend, reaching image qualities of 2.5 ± 0.5 for 1 min, 2.6 ± 1.0 for 2 min, 3.6 ± 0.9 for 3 min, 4.1 ± 1.0 for 4 min, 4.5 ± 0.5 for 6 min, 4.3 ± 0.7 for 8 min, and 4.3 ± 0.7 for 10 min (n = 7 patients) (**Fig 1**).

**Fig 2:**
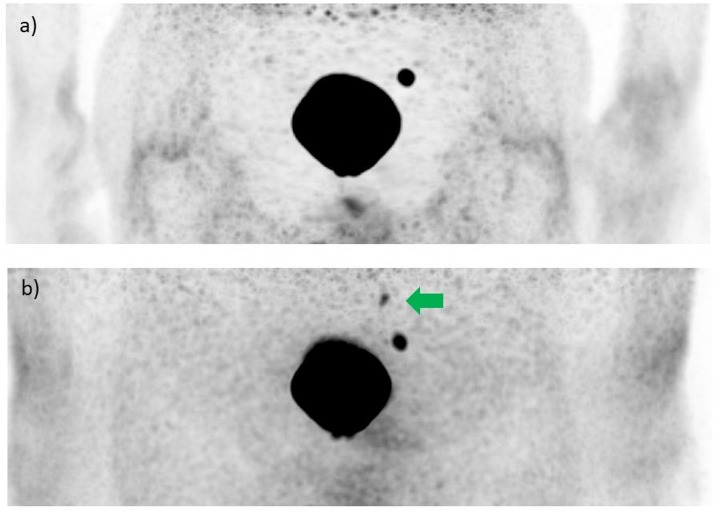
PET images of a patient (age: 66 years, serum PSA: 2.3 ng/ml) with recurrent PCa in two iliacal lymph nodes obtained with the PET/MR hybrid imaging system at 134 min after intravenous injection of ^68^Ga-HBED-CC-PSMA (146 MBq). a) Scatter und attenuation corrected PET images with intense ‘halo artifact’ showing only one iliacal lymph node. b) Non-scatter and attenuation corrected PET image showing both lymph nodes that were seen on PET/CT as well.

The image reconstruction algorithm of the PET/MRI system frequently produced artificially reduced uptake around the urinary bladder which was previously described as ‘halo artifact’ and might hamper the detection of lesions located in the vicinity of the genitourinary tract. In 18/20 (90%) of the patients, a halo artifact was observed, while in 2/20 (10%) of the patients no halo artifact was observed at any reconstructed PET image acquisition times. The presence of the halo artifact was classified by a scoring system from 1 (not present)– 5 (very intense halo artifact). The presence of the halo artifact linearly increased with rising PET image acquisition time, reaching scores of 1.2 ± 0.5 for 1 min, 2.0 ± 1.0 for 2 min, 2.5 ± 1.0 for 3 min, 3.2 ± 1.0 for 4 min, 3.4 ± 1.1 for 6 min, 3.8 ± 1.2 for 8 min, and 3.9 ± 1.4 for 10 min (n = 20 patients) (**Fig 1**). In three patients, a small lymph node lesion which was visualized with PET/CT acquired prior to PET/MR imaging was not detected with the scatter corrected PET images of the PET/MRI system due to this halo artifact (**Fig 2**). Two of these lesions were not detectable at acquisition times of 6 min and longer, one lesion was not detectable at all acquisition times.

**Fig 3:**
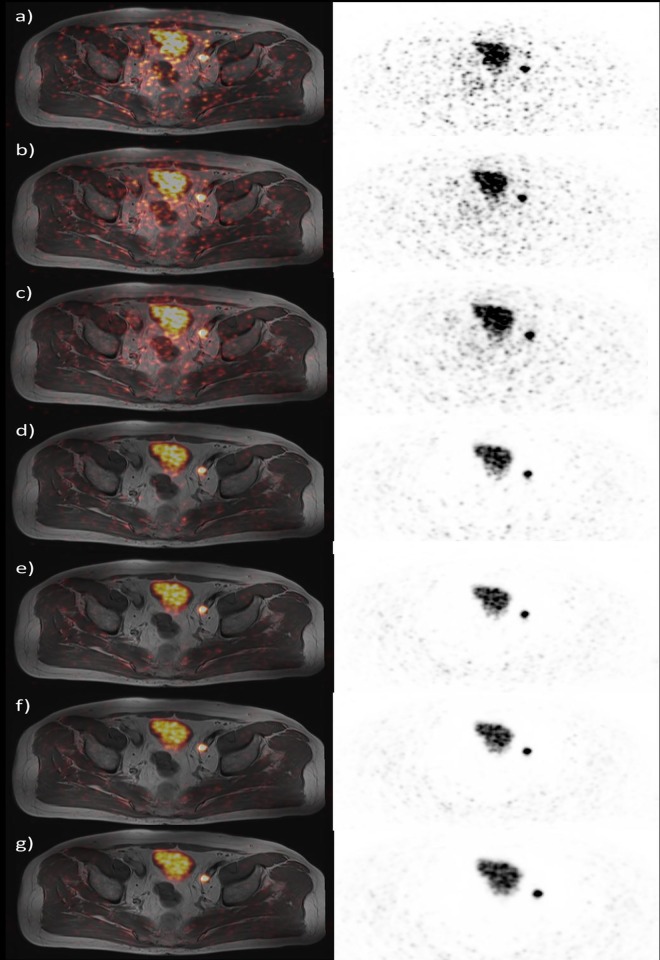
Image quality of ^68^Ga-HBED-CC-PSMA PET/MRI of a patient with recurrent PCa in an ilical lymph node. Images were acquired at 3 h p.i.. Left panel: PET/MR fusion image, right panel: PET image of the PET/MR hybrid imaging system. Acquisition times were a) 1 min, b) 2 min, c) 3 min, d) 4 min, e) 6 min, f) 8 min, and g) 10 min. PET image quality rises with increasing acquisition times and reaches a plateau at an acquisition time of 4 min. The halo artifact first occurs at image acquisition times of 4 min.

However, these tumor lesions were clearly visualized on the non-scatter corrected images. Still, the use of non-scatter corrected PET images limits image analyses to a qualitative fashion, quantitative analyses are not reliably possible. Outside of this artifact region, PET images derived from the PET/MRI system provided diagnostic image quality without further artifacts. Overall, PET image quality rises with increasing acquisition times and reaches a plateau at an acquisition time of 4 min (**Fig 3**).

### Quantitative analysis

**Fig 4:**
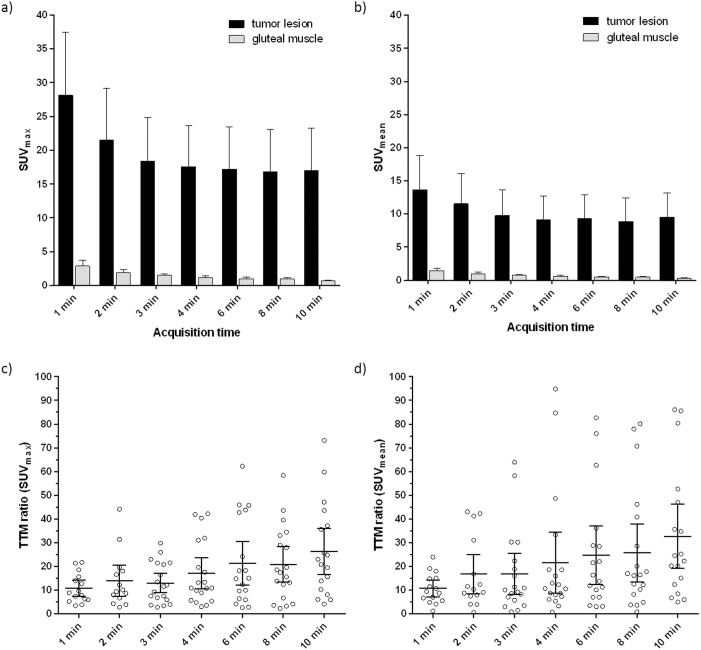
SUV_max_ (a)) and SUV_mean_ (b)) of tumor lesions and gluteal muscles at different PET acquisition times obtained with of ^68^Ga-HBED-CC-PSMA PET/MRI. SUV_max_ and SUV_mean_ show a decreasing trend with increasing acquisition times, but remain stable at acquisition times of 4 min and later. Tumor-to-muscle (TTM) ratios at different acquisition time points based on SUV_max_ (c)) and SUV_mean_ (d)) measurements. Data are represented in mean ± 95% confidence interval.

**Fig 5:**
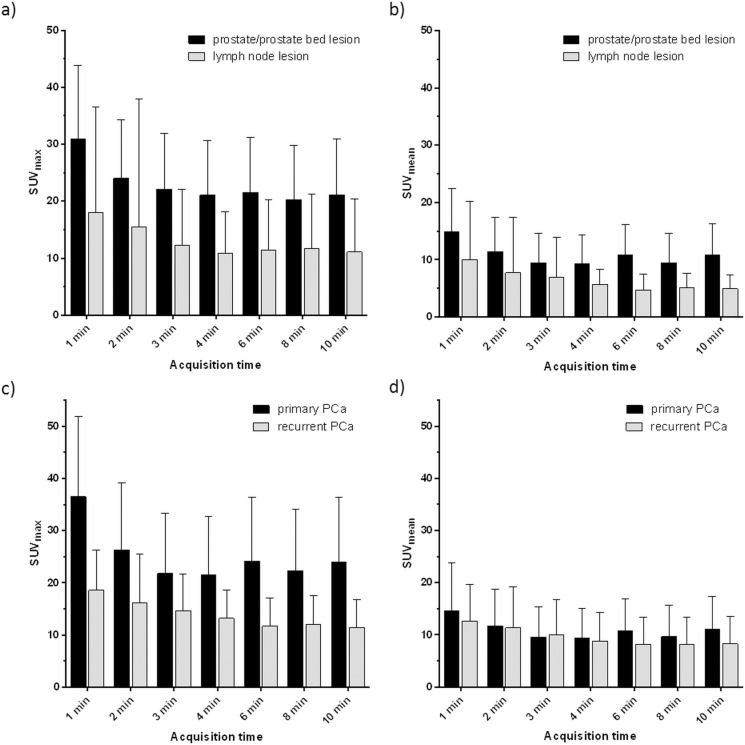
SUV_max_ (a) and c)) and SUV_mean_ (b) and d)) of prostate/prostate bed and lymph node lesions (upper panel) and primary and recurrent PCa (lower panel) at different PET acquisition times obtained with of ^68^Ga-HBED-CC-PSMA PET/MRI. SUV_max_ and SUV_mean_ show a decreasing trend with increasing acquisition times, but remain stable at acquisition times of 4 min and later. Data are represented in mean ± 95% confidence interval.

Overall, SUV_max_ and SUV_mean_ linearly decreased with increasing acquisition time, reaching a plateau at 4 min (mean 17.6, range 5.2–41.6 and mean 9.1, range 3.5–26.7, for SUV_max_ and SUV_mean_, respectively) (**Fig 4**). In patients with primary PCa and patients with recurrent disease, the same trend was observed (**Fig 5**). Tumor-to-muscle ratios increased with rising acquisition time from 10.8 (range 3.5–21.8) at 1 min acquisition time to 26.4 (range 5.9–59.8) at 10 min acquisition time for SUV_max_ measurements and from 10.7 (range 1.2–23.9) at 1 min acquisition time to 32.7 (range 5.0–52.7) at 10 min acquisition time for SUV_mean_ measurements (**Fig 4**).

## Discussion

The aim of this study was to optimize PET acquisition time in PET/MR imaging of primary and recurrent PCa using ^68^Ga-labeled HBED-CC-PSMA ligands. To assess image quality and lesion detectability, both visual and ROI-based quantitative approaches were used. Moreover, the SUV_max_ and SUV_mean_ of lesions with high PSMA ligand uptake served as measures of quantifiability.

So far, preliminary data have suggested the usefulness of ^68^Ga-HBED-CC-PSMA-PET/MR imaging for the detection of recurrent PCa [21]. It has been reported that ^68^Ga-HBED-CC-PSMA-PET/MRI was able to detect recurrent PCa accurately and with less irradiation exposure compared to PET/CT with this tracer. However, artifacts around the urinary bladder were observed in the PET component of the PET/MR images, which might hamper the visualization of tumor lesions in the vicinity of the urinary tract. To prevent the occurrence of such artifacts and to improve image quality, knowledge of optimal scanning procedures and image acquisition times is mandatory. So far, the role of variations in PET acquisition time on PET image quality and detectability of PCa lesions has not been evaluated, yet. Therefore, we characterized the influence of different acquisition times of ^68^Ga-HBED-CC-PSMA PET/MRI on PET image quality and detectability of PCa lesions in terms of SUV_max_/SUV_mean_.

In the present study, PET image quality increased with rising PET acquisition times, reaching a plateau at acquisition times of 4 min and longer for primary as well as for recurrent PCa. Lesion-based analysis revealed the same trend for tumor lesions of the prostate and prostate bed as well as for lymph node lesions. As expected, shorter image acquisition times were accompanied by increased image noise as an estimate of objective image quality. However, average subjective image quality was still classified with a quality of sufficient for the shortest acquisition time, rising to high image quality at PET acquisition times of only 4 min per bed position. This finding is in line with the tumor lesion detectability, which increased linearly reaching a maximum at PET acquisition times of 4 min. At acquisition times longer than 4 min, a slight decrease in tumor lesion detectability was observed, which was due to the occurrence of reduced PET signal intensity around the urinary bladder. Quantitative analysis of PET images obtained with the PET/MRI hybrid system revealed an inverse trend between SUV_max_/SUV_mean_ and rising PET acquisition times. At acquisition times of 4 min and longer however, a plateau was reached.

In the present study, diagnostic image quality was provided by the MR imaging component of the PET/MR hybrid imaging system in all patients, which is in line with the previous study [21]. However, the PET component frequently produced reduced signal intensity around the urinary bladder, which was previously described as ‘halo artifact’ [21] and restricted the detection of small iliacal lymph node lesions in three patients in the area of the artifact. However, this phenomenon was exclusively observed in the scatter corrected PET images. Analysis of the non-scatter corrected PET images obtained from the PET/MRI system revealed all tumor lesions which could not be visualized in the scatter corrected images due to the ‘halo artifact’. While quantitative analysis of these lesions is using the non-scatter corrected PET images of PET/MR imaging system is limited, all tumor lesions could at least be detected and visualized with PET/MRI. To ensure visualization of all tumor lesions by PET/MRI, we recommend careful analysis of both corrected and non-corrected PET images. Several strategies were hypothesized to influence the occurrence of this artifact, such as sufficient hydration of the patients, however, no direct correlation of the presence of the ‘halo artifact’ to the extent hydration of the patients could be identified. Since the presence of the halo artifact increases with rising PET image acquisition time, the occurrence of this artifact might be related to filling of the urinary bladder during scanning procedure.

Overall, these results indicate that PET image quality obtained with PET/MRI using ^68^Ga-labeled HBED-CC-PSMA ligands reaches its maximum around an acquisition time of 4 min while SUV_max_ and SUV_mean_ do not change significantly beyond this time point. Consequently, patients might profit from shorter scanning times. In addition, reduced scanning times will prevent extensive filling of the urinary bladder of the patients, which might help reduce the occurrence of artifacts due to high tracer accumulation in the urine. One limitation of the study is the fact that the optimization of image acquisition is specific to the PET/MR hybrid imaging system used here. For other PET/MR imaging systems, the optimal image acquisition time might differ slightly.

Another limitation of this study is the fact that, due to delays in clinical routine, tracer accumulation times differed between the examined patients. Ideally, tracer accumulation times should be the same for all patients.

In future, optimization of image acquisition procedures should be expanded to other PET/MR imaging systems. Moreover, it should be evaluated how the ‘halo artifact’ could be reduced and the exact influence of this artifact on the detectability of tumor lesions should be addressed.

## Conclusion

The optimal acquisition time of ^68^Ga-HBED-CC-PSMA-ligand PET/MRI in patients with primary and recurrent PCa was identified to be 4 min per bed position. At this acquisition time, PET image quality reaches its maximum while SUV_max_ and SUV_mean_ do not change significantly beyond this time point.

## Supporting Information

S1 File Supporting information is provided in the supporting information file ‘S1_file.pzfx. (PZFX)
